# miR-195-5p Inhibits Colon Cancer Progression via KRT23 Regulation

**DOI:** 10.3390/pharmaceutics16121554

**Published:** 2024-12-04

**Authors:** Emanuele Piccinno, Viviana Scalavino, Nicoletta Labarile, Raffaele Armentano, Gianluigi Giannelli, Grazia Serino

**Affiliations:** National Institute of Gastroenterology S. De Bellis, IRCCS Research Hospital, Via Turi 27, 70013 Castellana Grotte, BA, Italy; emanuele.piccinno@irccsdebellis.it (E.P.); viviana.scalavino@irccsdebellis.it (V.S.); nicoletta.labarile@irccsdebellis.it (N.L.); raffaele.armentano@irccsdebellis.it (R.A.); gianluigi.giannelli@irccsdebellis.it (G.G.)

**Keywords:** miR-195-5p, keratin 23, cytokeratins, colorectal cancer

## Abstract

Background/Objectives: KRT23 was recently discovered as an epithelial-specific intermediate filament protein in the type I keratin family. Many studies have underlined keratin’s involvement in several biological processes as well as in the pathogenesis of different diseases. Specifically, KRT23 was reported to affect the structural integrity of epithelial cells and to trigger cellular signaling leading to the onset of cancer. The aim of this study is to characterize a novel mechanism based on miR-195-5p/KRT23 in colorectal cancer. Methods: KRT23 mRNA and protein expression were characterized in FFPE sections from patients with CRC. The effects of miR-195-5p on KRT23 expression at the mRNA and protein levels were assessed by transient transfection experiments with mimic and inhibitor molecules. Cell attachment/detachment, migration, invasion, clone formation, and apoptosis were evaluated in human CRC cell lines after miR-195-5p mimic transfection. Results: We identified KRT23 as a putative target of miR-195-5p, a microRNA that we previously demonstrated to be reduced in CRC. We have proved the KRT23 expression deregulation in the tumoral section compared to adjacent normal mucosa in patients with CRC, according to the data derived from the public repository. We proved that the gain of miR-195-5p decreased the KRT23 expression. Conversely, we demonstrated that the inhibition of miR-195-5p led to an increase in KRT23 expression levels. We have demonstrated the in vitro effectiveness of miR-195-5p on CRC progression and that the in vivo intraperitoneal delivery of miR-195-5p mimic lowered colonic KRT23 mRNA and protein expression. Conclusions: These findings highlight a new regulatory mechanism by miR-195-5p in CRC affecting the keratin intermediate filaments and underline the miR-195-5p potential clinical properties.

## 1. Introduction

The three-dimensional organization of animal tissues is provided by the cytoskeleton, a dynamic system of interlinking protein filaments embedded within the cytoplasm of eukaryotic cells connecting to the neighboring cells, determining the cell shape and coordinating cellular movement [1]. The cytoskeleton’s ability to integrate the cells within the external environment and to transport cargoes within the cells is closely linked to several biological processes. To achieve these functions, the component polymers and regulatory proteins of the cytoskeleton are in constant flux and coordinate a multitude of cytoplasmic activities [2,3].

The three major protein families that form the filamentous structures of the cytoskeleton are microfilaments composed of several actin isoforms, microtubules consisting of α- and β-tubulin, and the intermediate filaments associated with molecular motors and regulatory protein complexes [4].

The keratins are the most abundant and typical intermediate filaments of epithelial cells subjected to continuous turnovers due to the exposition of epithelia to multiple forms of stress. As a result of the renewal and repair of the epithelia, the cytoskeleton organization is dynamically remodeled to ensure the cycle of proliferation, migration, and differentiation and the characteristic functions of epithelial cells [5].

Keratins include a large group of acidic and basic-to-neutral isotypes divided into 28 type I and 26 type II, respectively, that, beyond determining the cell mechanics and cohesion, act as targets and effectors of chemical signals interacting with major signaling pathways in an isoform-specific manner [6]. In this way, the specific keratin expression and structure affect cell adhesion, migration, and growth [7]. An alteration in keratin expression leads to pathological conditions such as colorectal cancer (CRC) [8].

Keratin 23 (KRT23) is an acidic type I keratin that was first identified in pancreatic cancer and subsequently was detected as a tumor-specific antigen in hepatocellular carcinoma (HCC) patients [9]. Since then, increasing evidence has shown the pro-cancer properties of KRT23 in certain tumors, although its role has not been comprehensively studied. The KRT23 amplification mediated by MYC promoted cell proliferation, inducing liver carcinogenesis [9]. In ovarian cancer, the KRT23 overexpression promoted cell migration and epithelial–mesenchymal transition (EMT) progression by regulating the TGF-β)/Smad pathway [10]. In addition, in colon cancer cells the upregulation of KRT23 affects the expression of crucial mediators that were involved in apoptosis and the cell cycle [11].

MicroRNA (miRNAs) are a family of small, single-stranded, non-coding RNA molecules that have a critical function in gene expression regulation at post-transcriptional levels. Physiologically, they bind to complementary mRNA sequences, affecting several biological processes through feedback mechanisms [12]. Since miRNAs are involved in development, differentiation, proliferation, apoptosis, and metabolic activity, any dysregulation of their expression could lead to the onset of many diseases [13,14,15,16,17,18]. Recently, several works have established the involvement of miRNAs in targeting specific genes that affect the intestinal barrier structure and function, triggering inflammatory response [15,16,17]. Similarly, we demonstrated the miR-195-5p impact in reversing the aberrant expression of PNN, a desmosome-associated protein involved in CRC development. Moreover, several miRNAs that are implicated in tumorigenesis altering the expression of keratin proteins have been identified.

miR-133a, miR-199a*, and miR-30-3p play a key role as tumor suppressors in bladder cancer, acting through a mechanism that underlines the transcriptional repression of KRT7 [19]. Downregulation of KRT16 by miR-365-3p increased the degradation of ß5-integrin and c-Met via a lysosomal cascade that led to the downstream repression of the Src/STAT3/FAK/ERK pathway and enhanced the chemosensitivity of oral squamous carcinoma cell lines towards 5-fluorouracil (5-FU) [20].

Considering the pathophysiological relevance of intermediate filament proteins of epithelial cells in tumorigenesis, the biological characterization of miRNAs abilities in the regulation of keratin could gain new knowledge in CRC pathogenesis and treatment.

In this work, we aimed to evaluate the involvement of KRT23 in colorectal cancer, focusing on its molecular regulation. We firstly demonstrated the KRT23 deregulation in patients with CRC. Then, we highlighted that miR-195-5p regulated the expression of KRT23 and functionally characterized the role of miR-195-5p in the CRC progression. These findings enhance the potential use of this miRNA in CRC treatment.

## 2. Materials and Methods

### 2.1. CRC Patients Tissues

Sixty formalin-fixed and paraffin-embedded (FFPE) human tissues with tumor and adjacent normal sections were obtained from 30 patients affected by CRC that were enrolled retrospectively in our institute in accordance with the following criteria: No one was subjected to therapy prior to the surgical resection, and thus patients with lower rectum cancer, who were undergoing therapy/treatment, were selectively excluded. Additionally, patients were selected without mutations. The research was performed following the principles of the Declaration of Helsinki. The study was approved by the local institutional ethics review board (Istituto Tumori Giovanni Paolo II, Bari, Italy n° 379/2020 of 16 September 2020). All participants signed a written informed consent. Tissue sections were used for hematoxylin and eosin staining and analyzed by a clinician to assess the propriety of the tissues and their phenotypical and/or pathological profiling.

### 2.2. RNA Isolation and RT-PCR of Human Sections

Tissues were incubated with a Deparaffinization Solution (Qiagen, Hilden, Germany). For total RNA isolation, we used the miRNeasy FFPE kit (Qiagen, Hilden, Germany) accordingly to the producer’s indications.

For KRT23 evaluation, retrotranscription was performed using SuperScript™ VILO™ MasterMix (Thermo Fisher Scientific, Bremen, Germany), and the RT-PCR amplification reactions were assessed on a CFX96 System (Biorad Laboratories, Hercules, CA, USA) with the SsoAdvanced Universal SYBR Green Supermix (BioRad Laboratories, Hercules, CA, USA) and the QuantiTect Primer Assay for GAPDH and KRT23 (Qiagen, Hilden, Germany). GAPDH gene was used as a reference standard to normalize the KRT23 expression. The 2^−ΔCt^ formula was used to calculate the KRT23 relative expression.

### 2.3. Immunohistochemistry (IHC)

Prior to immunostaining, FFPE tissues were cut into three μm sections and mounted on Apex Bond IHC slides (Leica Biosystems, Buffalo Grove, IL, USA). The specimens were deparaffinized and the immunohistochemical staining was carried out in the BOND III automated immunostainer (Leica Biosystems, Buffalo Grove, IL, USA) using the anti-KRT23 primary antibody (ab156569, Abcam, Cambridge, UK; 1:100 dilution). Sections were then counterstained with hematoxylin. For antigen retrieval, the BOND Epitope Retrieval Solution 2 (Leica Biosystems, Buffalo Grove, IL, USA) with citrate buffer pH 6 was used. The Bond Polymer Refine Detection Kit (Leica Biosystems, Buffalo Grove, IL, USA) was employed as a chromogen reagent. IHC scoring was performed by a pathologist on the basis of extensiveness and intensity of biomarker as follows: 0, (no staining) negative; 1, weak expression; 2, moderate expression; and 3, strong expression.

### 2.4. Cell Culture

HCT116 and HT29 colonic epithelial cell lines were acquired from ATCC (American Type Culture Collection, Manassas, VA, USA) and grown in Dulbecco’s Modified Eagle Medium (DMEM, Thermo Fisher Scientific, Waltham, MA, USA) using 10% heat-inactivated fetal bovine serum (FBS, Thermo Fisher Scientific, Waltham, MA, USA), 1% streptomycin/penicillin (Thermo Fisher Scientific, Waltham, MA, USA), 10 mM HEPES (Sigma-Aldrich, St. Louis, MO, USA), and 1 mM sodium pyruvate (Sigma-Aldrich, St. Louis, MO, USA) as supplements.

For RNA and protein extraction, cells were seeded in 12-well and 6-well plates, respectively. HT29 and HCT116 were transfected with molecules of miR-195-5p mimic or inhibitor (Life Technologies, Carlsbad, CA, USA) at concentrations of 30 nM and 50 nM using TKO transfection reagent (Mirus Bio LLC, Madison, WI, USA) once they reached the confluence. For attachment/detachment and clone formation assay, cells were plated in 6-wells before transfection.

### 2.5. RNA Extraction and RT-PCR of CRC Cell Lines

Mice tissues and cell cultures were lysed with TRIzol reagent (Invitrogen by Thermo Fisher Scientific, Waltham, MA, USA) to extract total RNA following the producer’s recommendations. The NanoDrop ND-2000 Spectrophotometer (Thermo Fisher Scientific, Waltham, MA, USA) was used to establish the concentrations of RNA samples.

For KRT23 quantification, the iScript Reverse Transcription Supermix (BioRad Laboratories, CA, USA) was used for reverse transcription according to the provider’s protocol. Real-time PCR amplification reactions were assessed in 20 μL of final volume on a CFX96 System (Biorad Laboratories, Hercules, CA, USA) using the SsoAd-vanced Universal SYBR Green Supermix (BioRad Laboratories, Hercules, CA, USA) and the QuantiTect Primer Assay for *KRT23* (Biorad Laboratories, Hercules, CA, USA) and *GAPDH* (Qiagen, Hilden, Germany).

The KRT23 and GAPDH expressions were carried out in four independent experiments. GAPDH gene amplification was used as a reference standard to normalize the relative expression of KRT23. The 2^−ΔΔCt^ formula was used to calculate the KRT23 relative expression.

### 2.6. Western Blot

Total protein from HCT116 and HT29 cell lines was obtained by means of T-PER Tissue Protein Extraction Reagent (Thermo Fisher Scientific, Waltham, MA, USA) with cocktail proteinase inhibitors (Sigma-Aldrich, St. Louis, MO, USA). The Bradford colorimetric assay (BioRad Laboratories, Hercules, CA, USA) was used to determine the protein concentration. For each sample, an equal amount of proteins was heat-denatured at 100 °C for 5 min in reducing Laemmli Sample Buffer (Biorad Laboratories, Hercules, CA, USA). Proteins were electroformed on 4–20% Mini-PROTEAN TGX Stain-Free Gels (Biorad Laboratories, Hercules, CA, USA) and transferred onto PVDF membranes (0.2 μm pore size) (Biorad Laboratories, Hercules, CA, USA). The primary and secondary antibodies were incubated in an automated iBind Flex Western Device (Thermo Fisher Scientific, Waltham, MA, USA), and the signal intensities were revealed using the Chemidoc System (Biorad Laboratories, Hercules, CA, USA). Blots were analyzed with Image Lab Software version 5.2.1 (Biorad Laboratories, Hercules, CA, USA) and quantified by ImageJ Software 1.54d. β-Tubulin expression was used to normalize the target protein signal.

Primary antibody of rabbit monoclonal anti-KRT23 (ab156569, Abcam, Cambridge, UK; 1:2000 dilution) and mouse monoclonal ß-Tubulin (sc-166729, Santa Cruz Biotechnology, Inc., Heidelberg, Germany; dilution 1:1000) as well as secondary antibodies of Goat Anti-mouse IgG-(H + L)-HRP conjugate (170-6516, Biorad Laboratories, CA, USA; dilution 1:500) and Goat Anti-rabbit IgG-(H + L)-HRP conjugate (#31466, Invitrogen, Carlsbad, CA, USA; dilution 1:2500) were used for western blot analysis.

### 2.7. Attachment and Detachment Assay

Forty-eight hours post-transfection, cells were harvested, counted, and replated in 24-well plates (5 × 10^4^/well) (Corning, Corning, NY, USA). For attachment assay, after 1 h of incubation at 37 °C, unattached cells were washed away. After trypsinization, the attached cell number was determined using trypan blue stain by means of the Countess™ 3 Automated Cell Counter (Thermo Fisher Scientific, Waltham, MA, USA).

For cell detachment assays, transfected cells (1 × 10^5^/well) were cultured in 24-well plates for 24 h and detached using 0.05% trypsin for 1 or 2 min. Then, the detached cells were collected and automatically counted while the remaining cells were re-incubated with 0.25% trypsin to detach and automatically counted. The data were presented as a percentage of the detached cells to total cells.

### 2.8. Clone Formation Assay

HCT116 and HT29 transfected cells were trypsinized and plated in 6-well plates (5 × 10^2^ cells/well). The cells were kept for two weeks at 37 °C with 5% CO_2_ in a humidified incubator to form colonies. The colonies formed were PBS washed, fixed with 4% paraformaldehyde for 15 min, and stained with crystal violet. After washing, images of the stained plates were captured, and the cell colonies that contained more than 50 cells were counted using ImageJ Software 1.54d. Each treatment was performed in triplicate.

### 2.9. Annexin V Staining

After transfection, HCT116 and HT29 cell lines were counted, stained with Muse Annexin V and Dead Cell Reagent (Cytek Biosciences, Fremont, CA, USA), and incubated at room temperature for 20 min in the dark. Apoptosis detection was performed with the Guava Muse Cell Analyzer (Luminex Corp, Austin, TX, USA) according to the provider’s directive.

### 2.10. Immunofluorescence in FFPE from Mice Tissue

Three μm sections of FFPE colon parts from mice were deparaffinized, and antigen retrieval was performed using citrate buffer pH 6 in a water bath at 98 °C for 30 min. Tissue slides were treated with a solution of 0.5% Triton X-100 in PBS 1× for 10 min at room temperature and then blocked in PBS + BSA 5% for 1 h at room temperature. Then, slides were incubated with primary antibody mouse cytokeratin 23 (sc-365892, Santa Cruz Biotechnologies, Inc., Heidelberg, Germany; dilution 1:100) overnight at 4 °C and then with secondary antibody chicken anti-mouse IgG (H + L) Alexa Fluor 488 (A-21200, Invitrogen, dilution 1:200) for 1 h. Slides were mounted with ProLong Gold Antifade Mountant with DAPI (Thermo Fisher Scientific) and a glass coverslip. Images were acquired with a fluorescence microscope (Eclipse Ti2, Nikon Inc., Melville, NY, USA) using filters for DAPI and FITC using a 20× objective lens.

### 2.11. Bioinformatic and Statistical Analysis

The GEPIA tool (http://gepia.cancer-pku.cn/index.html, accessed on 31 July 2023 [21]) was exploited to evaluate KRT23 expression in tumoral and normal tissue.

miR-195-5p gene targets were predicted with the miRwalk algorithm (http://bioinfo.univ-rouen.fr/mirabel/; accessed on 25 September 2023 [22]). Data were analyzed using GraphPad Prism software version 9.0.0 and are shown as mean  ±  SEM. Statistical significance of data obtained from at least three independent experiments was analyzed with a two-tailed Student’s *t*-test. The difference between experimental conditions was statistically significant at *p* < 0.05.

## 3. Results

### 3.1. KRT23 as a Target of miR-195-5p

**Figure 1:**
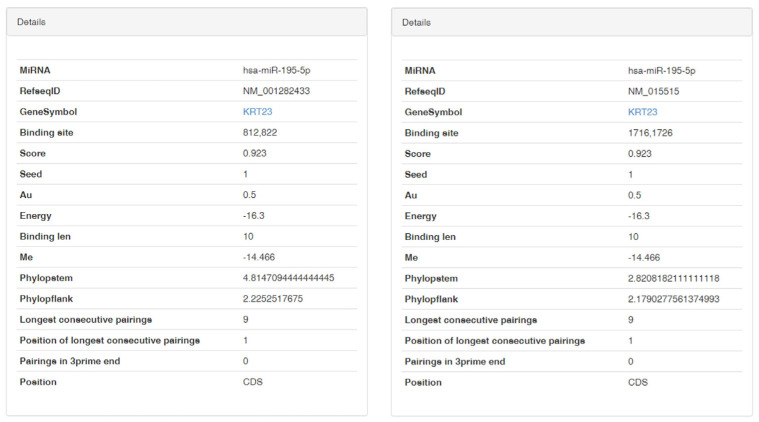
miR-195-5p targets *KRT23* binding two specific sequences in CDS mRNA. miRwalk algorithm was used for the analysis founding that miR-195-5p links *KRT23* CDS in two different sites.

In our recent works, we investigated the miR-195-5p involvement in CRC [14,23,24]. To further assess the molecular mechanism regulated by miR-195-5p in the CRC development, we conducted a bioinformatic analysis to predict its potential target genes, focusing our attention also on the 5’ untranslated region (UTR) and the coding sequence (CDS) regulatory regions. The bioinformatic analysis highlighted that miR-195-5p has two putative binding sites located in the CDS region of the *KRT23* gene (Figure 1). This gene is essential to guaranteeing tissue homeostasis, and its aberrant expression may lead to the onset of CRC.

### 3.2. KRT23 Expression in Patients with CRC

**Figure 2:**
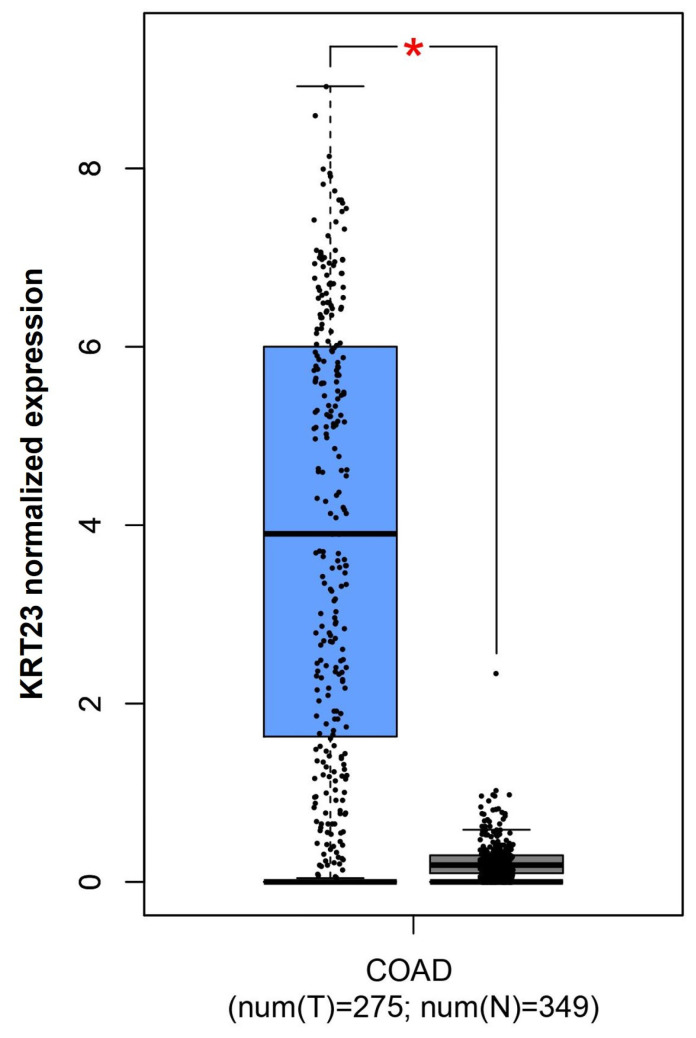
*KRT23* expression in CRC. GEPIA tool investigation highlighted a statistically significant deregulation of *KRT23* between tumor and healthy control status. * *p* < 0.01.

In order to evaluate the *KRT23* expression in patients with CRC, we used the TCGA and GTEx databases containing public cancer sequencing data. The analysis performed by the GEPIA tool underlined that CRC tissue has more expression of *KRT23* gene expression than adjacent tissue (*p* < 0.01; Figure 2) [21].

To further investigate the deregulation of *KRT23* in CRC, we analyzed the KRT23 expression levels in our cohort of 30 patients with CRC. For each patient we compared the tumor expression value with those of the corresponding adjacent normal sections. Demographic and clinical characteristics of patients are summarized in Table 1.

**Figure 3:**
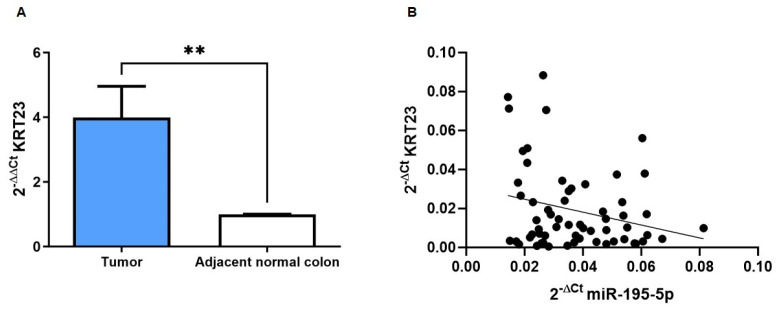
*KRT23* expression in patients with CRC (n = 30). (**A**) The RNA was extracted to perform qPCR analysis starting from FFPE tissue blocks that include tumor and adjacent normal colon. Our results demonstrated the crucial increase in expression of *KRT23* in CRC tissue. ** *p* < 0.001. (**B**) Pearson correlation in CRC tissues demonstrated a negative correlation between miR-195-5p and *KRT23* expression, indicating that miR-195-5p may be involved in the regulation of *KRT23*. r = −0.249, *p* < 0.05. Each point represents a sample.

Data from real-time PCR confirmed the aberrant overexpression of *KRT23* in tumoral sections when compared to adjacent normal tissues (*p* < 0.001; Figure 3A). In addition, we demonstrated a negative correlation between *KTR23* and miR-195-5p expression previously analyzed on the same samples [22] (r = −0.249, *p* < 0.05; Figure 3B). This negative correlation indicates that miR-195-5p may regulate KRT23.

**Figure 4:**
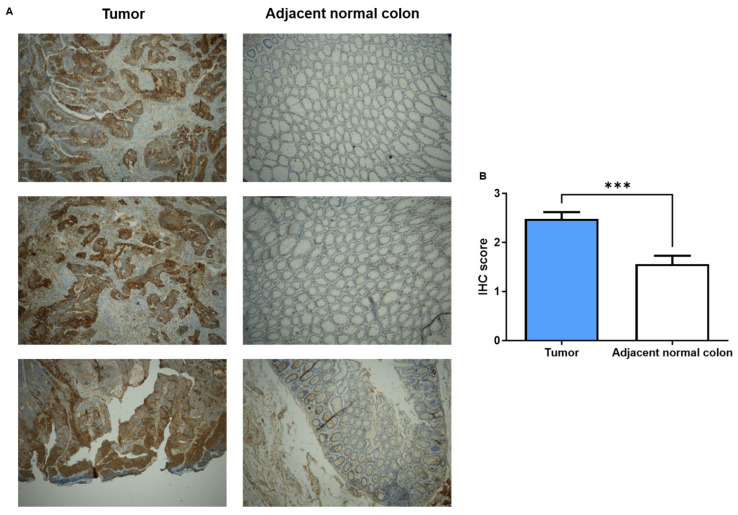
KRT23 protein expression at IHC in FFPE tissues of patients with CRC. (**A**) Representative images acquired at 4× original magnification showing a strong stain in CRC tissue compared to adjacent normal mucosa. (**B**) The immunoreactivity score quantified the signal intensity that corresponds to the KRT23 expression in colonic epithelium *** *p* < 0.0001.

To clearly validate the aberrant expression of KRT23 in CRC, we assessed the KRT23 protein levels by immunohistochemistry (IHC). The examination was carried out on the tissues derived from the same patients with CRC used in the RNA analysis. In accordance with the data obtained by qPCR, the IHC staining underlined the increased levels of KRT23 in the tumoral section compared to the corresponding peritumoral sample (Figure 4A). We also defined an immunoreactivity score from 0 to 3 based on positivity staining intensity (*p* < 0.0001, Figure 4B). The score reported in Figure 4B demonstrated once more the deregulation of KRT23 in patients with CRC.

### 3.3. miR-195-5p Modulates KRT23 Expression

**Figure 5:**
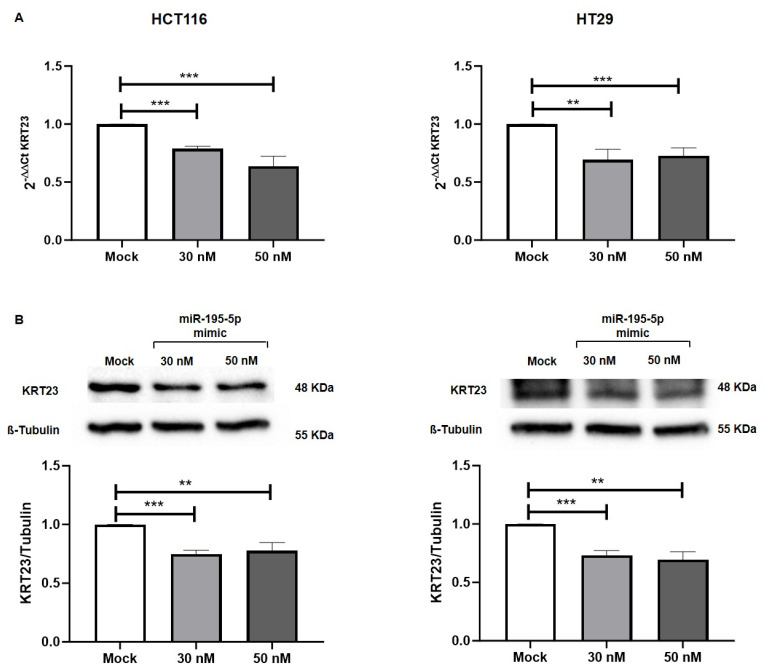
KRT23 expression in mimic transfected cell lines. (**A**) KRT23 gene expression was assessed in HCT116 (**left**) and HT29 (**right**) transfected cells. The raise of miR-195-5p at 30 nM and 50 nM concentrations by transient transfection led to a valuable reduction of KRT23 in both cell lines. Expression data were normalized on the Gapdh housekeeping gene. Graphs are representative of four independent experiments (mean ± SEM). ** *p* < 0.001; *** *p* < 0.0001. (**B**) KRT23 protein expression in mimic transfected cells. The increase in intracellular levels of miR-195-5p induced an effective decrease in KRT23 levels in both cell lines. KRT23 values were obtained by dividing the normalized transfected sample values by the normalized mock-control sample values. Normalization was performed on the values of β-tubulin housekeeping protein. Supplementary File S1 contained the raw data for all Western blot experiments. ** *p* < 0.001; *** *p* < 0.0001.

In order to evaluate if miR-195-5p was able to modulate the KRT23 expression levels, we carried out transient transfection with synthetic compounds of miR-195-5p mimic at the concentrations of 30 nM and 50 nM in vitro. The gain of function of miR-195-5p in HCT116 and HT29 cell lines showed a significant reduction of *KRT23* at mRNA levels at both mimic concentrations (*p* < 0.001; Figure 5A).

In the same way, enhancing the miR-195-5p intracellular level, we examined whether miR-195-5p influenced the KRT23 protein expression. Even in this case, the miR-195-5p transfection at 30 nM and 50 nM was also able to markedly decrease the KRT23 protein expression level in transfected cells compared with mock control. (*p* < 0.001; Figure 5B).

**Figure 6:**
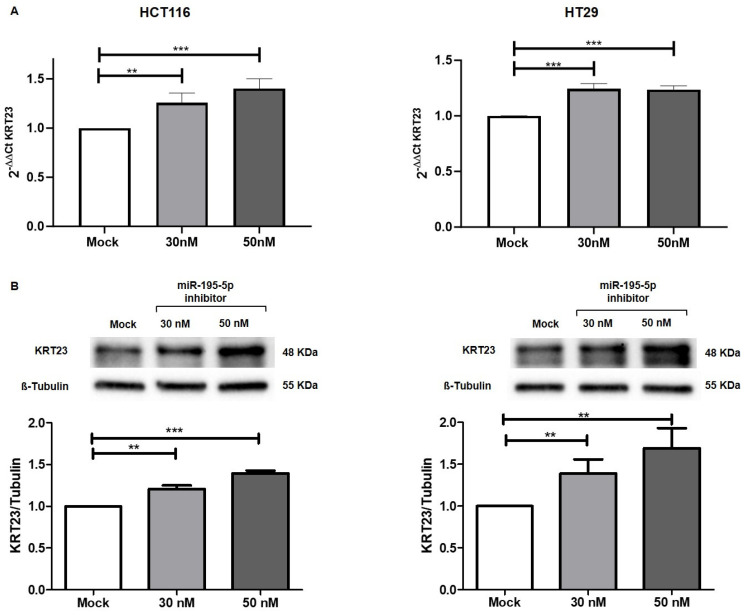
KRT23 expression after miR-195-5p inhibitor transfection. In HCT116 and HT29 cells, KRT23 mRNA (**A**) and protein expression (**B**) levels were significantly increased after transfection compared to mock control confirming the KRT23 regulation by miR-195-5p. mRNA expression data were normalized on the GAPDH housekeeping gene. KRT23 protein values were obtained by dividing the normalized transfected sample values by the normalized mock-control sample values. Data were normalized on the values of β-tubulin housekeeping protein. Supplementary File S1 contained the raw data for all Western blot experiments. ** *p* < 0.001; *** *p* < 0.0001.

In the reverse experiment, to additionally confirm the involvement of miR-195-5p in the regulation of KRT23 expression, we transiently transfected the CRC cell lines with molecules of miR-195-5p inhibitor. The decrease in intracellular levels of miR-195-5p in HCT116 and HT29 leads to a significant increase in KRT23 at mRNA levels (*p* < 0.01; Figure 6A). Interestingly, the inhibition of miR-195-p was able to upregulate the KRT23 protein expression levels (*p* < 0.01; Figure 6B). Altogether, these data confirm that miR-195-5p is responsible for the regulation of KRT23 expression levels.

### 3.4. miR-195-5p Affects Attachment and Detachment of CRC Cell Lines

**Figure 7:**
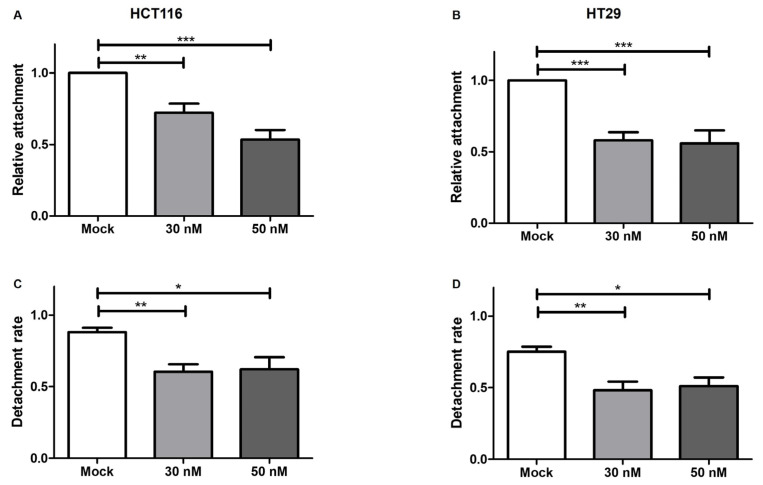
miR-195-5p mimic inhibits cell attachment and detachment. (**A**,**B**) Attachment assay employed on HCT116 (**left**) and HT29 (**right**) after mimic transfection. In transfected conditions, the relative attachment of cells was found to be deeply reduced compared to mock control. The values were obtained by dividing the values of the normalized transfected sample with the normalized mock-control sample and derived at least from four independent experiments. ANOVA *p* < 0.0001 (**C**,**D**) Detachment rate of HCT116 and HT29 following mimic transient transfection. The intracellular increase level of miR-195-5p affected the detachment capacity of transfected cells. The data were presented as a percentage of the detached cells to total cells and are presented as the mean  ±  SEM of at least four independent experiments. ANOVA *p* < 0.001 * *p* < 0.05, ** *p* < 0.001, *** *p* < 0.0001.

In order to evaluate the potential clinical properties of miR-195-5p in the CRC suppression, we studied the influence of the miR-195-5p increment in attachment and detachment assays. The raise of miR-195-5p in HT29 and HCT116 induced a strong inhibition of the attachment/detachment ability in transfected conditions (ANOVA *p* < 0.0001; ANOVA *p* < 0.001; Figure 7).

In addition, we have assessed the migration and invasion ability of HCT116 and HT29 cells. According to cell attachment/detachment results, the transwell assay confirmed that the miR-195-5p gain markedly suppresses the migration and invasion rate of CRC cells, suggesting the positive effects of miR-195-5p in enhancing the assembly and function of cell adhesion (Figure S1).

### 3.5. miR-195-5p Prevents the Colony Formation

**Figure 8:**
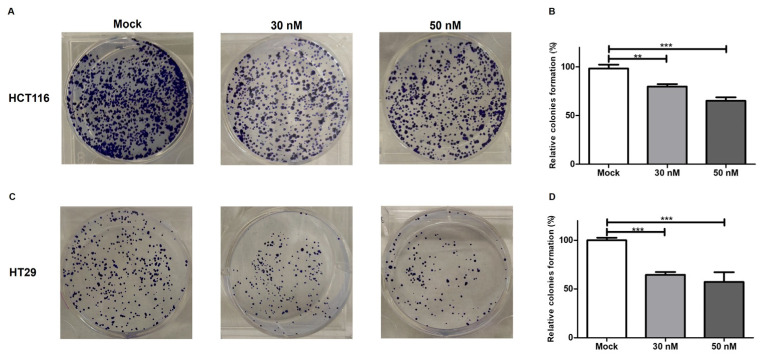
miR-195-5p mimic effectiveness on the formation of HCT-116 cell (**A**,**B**) and HT29 cell (**C**,**D**) colonies. Transient transfection with molecules of miR-195-5p markedly decreased the cell proliferative rates. After transfection, cell clonogenic potential was significantly declined. For each cell line, the representative images of the colony formation assay and the relative proliferation rate are shown. Magnification 4×. The results are presented as the mean of at least four independent experiments ± SEM. ANOVA *p* < 0.0001; ** *p* < 0.001; *** *p* < 0.0001.

To further define the contribution of miR-195-5p in CRC progression, we investigated its effect on CRC cell line clone formation ability. We overexpressed miR-195-5p in HT29 and HCT116, highlighting its function in suppressing the clonogenic potential of CRC cells. The induced expression of mature miR-195-5p at 30 nM and 50 nM concentrations resulted in a significant repression of the cell proliferation compared to untransfected conditions, suggesting the protective role of miR-195-5p in CRC (ANOVA *p* < 0.0001; Figure 8).

### 3.6. miR-195-5p Induces Apoptosis

To further evaluate the functional effects of miR-195-5p, we overexpressed miR-195-5p molecules in HCT116 and HT29 cell lines and examined their effect on cellular apoptosis. After transfection, we observed significant differences in the percentage of live cells and total apoptotic cells between transfected cells and mock controls in all cell lines (*p* < 0.05, Figure S2A,B). Specifically, we found that raising miR-195-5p intracellular levels at 30 nM and 50 nM concentrations increased the proportions of total apoptotic cells that include early and late apoptotic cells (Figure S2C,D).

### 3.7. In Vivo Determination of KRT23 Expression

In our recent study, we have elucidated the effectiveness of miR-195-5p, assessing a CRC mouse model induced by administration of azoxymethane (AOM)/dextran sodium sulfate (DSS) [24]. In brief, we analyzed two groups of male C57BL/6 mice divided into a control group (vehicle, n = 14) and a treated group (miR-195-5p mimic, n = 14). In each animal, cycles of AOM and DSS were administered, followed by a cycle of 15 days of water. Until sacrifice, miR-195-5p mimic and vehicle were intraperitoneally injected twice a week.

**Figure 9:**
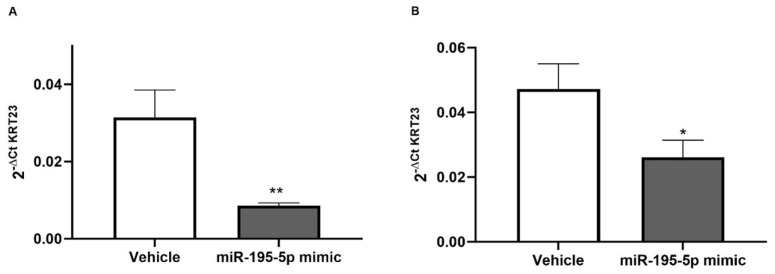
Impact of miR-195-5p mimic administration on *Krt23* expression in CRC mice model (n = 14 mice/group). *Krt23* expression in both medial (**A**) and distal colon segments (**B**) was significantly decreased in the miR-195-5p-treated group as compared with vehicle mice. Expression data were normalized on housekeeping gene *Gapdh* and are shown as mean ± SEM. * *p* < 0.01, ** *p* < 0.001.

Furthermore, to characterize the molecular mechanisms controlled by miR-195-5p in AOM/DSS mice models, we examined the *KRT23* expression in colon segments of mice obtained in our previous in vivo experiments [24]. *Krt23* levels were found to be clearly reduced in medial as well as in distal colons of mice handled with intraperitoneal administration of miR-195-5p (*p* < 0.001; Figure 9).

**Figure 10:**
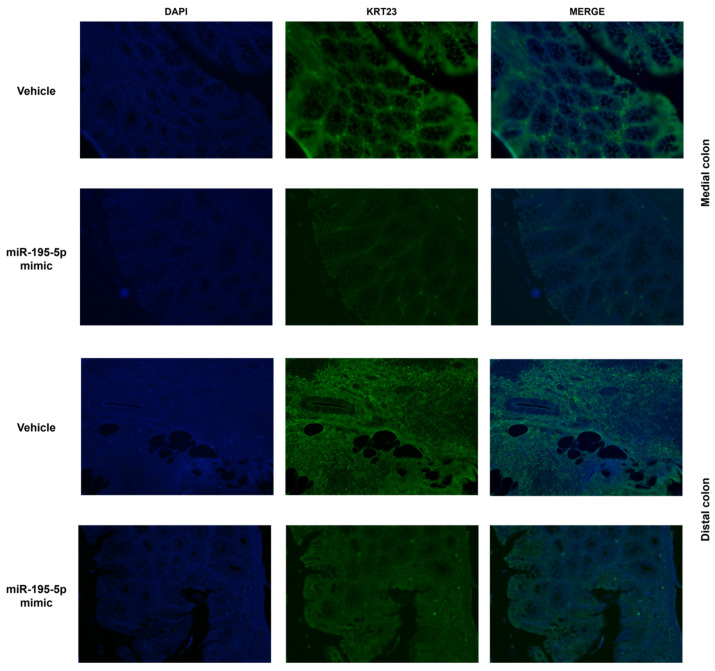
miR-195-5p in vivo effect on KRT23 protein expression in AOM/DSS mice (n = 14 mice/group). According to mRNA data, KRT23 protein expression in the medial and distal colons was strongly reduced in miR-195-5p-treated mice compared to the vehicle group. Each single-channel image derived from DAPI and KRT23 signals is merged. Original magnification: 20×.

Moreover, we also investigated the expression of KRT23 protein in the same colon tissue used for mRNA analysis. KRT23 protein levels were strongly decreased in the medial and distal colons of mice after miR-195-5p mimic administration (Figure 10). All these findings enhanced the suggestion of KRT23 and miR-195-5p as effective good candidate targets for CRC therapy.

## 4. Discussion

The cell adhesion mechanisms involved in the connections to the internal cytoskeleton determine the tissue’s architecture and function. The cytoskeleton impacts the cells pivotal functions through dynamic changes that provide structural support and organization, ensuring cell shape change and movement, guaranteeing mechanical resistance to stress, and integrating external stimuli [25].

Keratins are epithelial-specific intermediate filament proteins that are generally classified as structural proteins, although in the last years several studies have recognized their active involvement in many cellular mechanisms, like protein synthesis, motility, cell growth, and signaling [26,27]. Keratins members have widely been exploited/employed for diagnostic purposes, but in the last few years, have been emerging as prognostic markers that are able to directly affect either epithelial tumorigenesis or treatment responsiveness [28,29].

The study concerning the multifactorial properties of keratins could facilitate the establishment of new therapeutic targets that allow earlier cancer diagnosis and more efficiency in cancer therapies [30,31]. In this context, KRT23 is a recently identified type I keratin component that guarantees the cell’s structural integrity and is crucial for several cellular functions, such as apoptosis [32,33]. Since epithelial cells have a specific and variable signature of cytokeratin during development and differentiation that is only partially affected by malignant transformation, KRT23 was identified as a specific circulating marker of HCC [34]. Other evidence has reported the KRT23-related mechanism of tumorigenesis in many types of tumors. In gastric cancer, KRT23 knockout inhibited the proliferation and arrested cell cycle progression through a reduced phosphorylation of ERK1/2 and p38 [33]. Kim and colleagues demonstrated that the induction of KRT23 mediated by MYC transcriptional potentiation promotes HCC growth [9]. Similarly, KRT23 overexpression facilitates the migration of ovarian tumor cells through EMT-regulating TGF-β/Smad signaling [10]. In CRC, the downregulation of KRT23 affected the expression of genes involved in DNA damage response, affecting the proliferation of CRC cells and their responsiveness to radiation [35]. In addition, overexpression of KRT23 induced the telomerase reverse transcriptase activity, promoting CRC cell migration [36].

Taken together, these studies argued the tumorigenic properties of KRT23 but its molecular mechanisms and potential regulators in CRC are still not clearly clarified and require in-depth exploration.

miRNAs are an emerging class of non-coding RNAs that regulate human gene expression and could function as modulators in multiple pathological and biological progressions, such as cancer cell differentiation, proliferation, and apoptosis [37,38]. Growing evidence reported miRNAs as clinical, diagnostic, and prognostic biomarkers, as well as treatment approaches highlighting their properties as either oncogenes or tumor suppressors by directly targeting mRNAs and participating in the mechanism of tumor occurrence and development [39]. In oral cancer cells, miR-485-5p suppresses keratin 17 (KRT17), which in turn represses the integrin β4/α6/FAK/Src/ERK/β-catenin pathway, impacting cancer progression and resistance to cisplatin and carboplatin [40]. Moreover, a recent study assessed that the overexpression of miR-642 decreases the proliferation and migration of gastric tumor cells affecting the KRT19 expression [41]. However, to date, investigations of miRNA involvement in CRC by regulating keratin members are still lacking.

Recently, we have demonstrated the miR-195-5p deregulation in patients with CRC and its critical potential role as a tumor suppressor [24]. Indeed, we have documented that the in vitro gain of function of miR-195-5p strongly repressed colon cancer cell proliferation, viability, migration, and invasion [23,24], regulating desmosome junction. In addition, the miR-195-5p in vivo administration markedly decreased the number of malignant formations in the entire colon of AOM/DSS-treated mice as well as affected tumor growth [24]. In this concern, we have further characterized a new miR-195-5p/PNN interaction involved in CRC development that affected the desmosome complex. In detail, we proved the miR-195-5p abilities reverse the overexpression of PNN in both in vitro and in vivo models. Moreover, PNN modulation by miR-195-5p indirectly increased the levels of KRT8 and KRT19, reinforcing the strength of adhesive junctions.

In this study, for the first time, we demonstrate a miR-195-5p/KRT23 axis. Starting from a bioinformatic analysis, we have determined that KRT23, a gene that encodes for a type I keratin protein, was a putative target of miR-195-5p. Consequently, we have examined the KRT23 expression in CRC using public cancer OMICS data from an online repository. Interestingly, the evaluation of KRT23 levels in tissue specimens collected in our institute of patients with CRC confirmed the KRT23 overexpression in the tumoral section compared to normal mucosa.

We have also proved that miR-195-5p increases in CRC cell lines manages the mRNA and protein expression of KRT23. On the other hand, we have also demonstrated that the inhibition of miR-195-5p in colonic cells increased the KRT23 expression, confirming the role of miR-195-5p on KRT23 regulation. Furthermore, with the aim of defining the potential effectiveness of miR-195-5p increase, we evaluated the cell attachment and detachment ability as well as cell clonogenic potential after miR-195-5p mimic transfection. Notably, after raising the intracellular level of miR-195-5p, we found that the cellular attach/detach potential, the migration/invasion abilities, and the relative proliferation rate were significantly suppressed. In vitro, miR-195-p impacted also on apoptosis process confirming its positive properties. Finally, we demonstrated that intraperitoneal delivery of miR-195-5p mimic lowered colonic KRT23 expression in the CRC mice model.

Our findings further highlighted the potential application of miR-195-5p, elucidating a novel regulatory mechanism of keratin intermediate filaments in CRC. However, future research will need to be conducted to clarify the molecular mechanisms related to KRT23 in CRC progression.

## 5. Conclusions

In conclusion, our results support the potential clinical properties of miR-195-5p by targeting the KRT23 expression. We have proved that the recently discovered KRT23 was markedly overexpressed in tumoral tissues of patients with CRC and that miR-195-5p can reverse the KRT23 dysregulation in both CRC cell lines and AOM/DSS-treated mice. These results enhance the clinical significance of miR-195-5p in CRC.

## 6. Patents

In Italy, a patent entitled “MiRNA or combination of miRNAs for therapy” was issued on 4 June 2024 to the Ente Ospedaliero Specializzato in Gastroenterologia “Saverio de Bellis”.

## Figures and Tables

**Table 1:** Demographic and clinical characteristics of patients with CRC.

Characteristic	Patients with CRC (n = 30)
*Sex*	
Female (%)	13 (44.4)
Male (%)	17 (56.6)
*Age at diagnosis*	
Mean ± SD	69.4 ± 12.9
*TNM stage*	
I (%)	1 (3.3)
II (%)	12 (40)
III (%)	17 (56.7)
IV (%)	0 (0)
*Nodal status*	
Positive (%)	18 (60)
Negative (%)	12 (40)

## Data Availability

Data are within the article, Supplementary Materials, and Supplementary File S1.

## Supplementary Materials

The following supporting information can be downloaded at https://www.mdpi.com/article/10.3390/pharmaceutics16121554/s1, Figure S1. Migration and invasion transwell assay in HCT116 and HT29 cell lines after miR-195-5p transfection. Figure S2. Apoptotic profile of HCT116 and HT29 after transfection. File S1: Raw Data of All Western Blot Experiments.
