# Supplementary material for: miR-195-5p Inhibits Colon Cancer Progression via KRT23 Regulation
Source: Pharmaceutics. 2024 Dec 4;16(12):1554. doi: 10.3390/pharmaceutics16121554 (PMC11680050; doi:10.3390/pharmaceutics16121554)
Supplement: Supplementary file 1 [file pharmaceutics-16-01554-s001.zip › File S1.pdf]

HCT116 Western blot for KRT23

Experiment 1 miR-195-5p mimic

KRT23

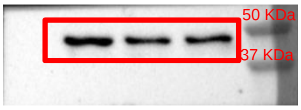

Lane 1 Mock
Lane 2 miR-195-5p mimic 30 nM
Lane 3 miR-195-5p mimic 50 nM
Lane 4 Standard Molecular Weight

ß-tubulin

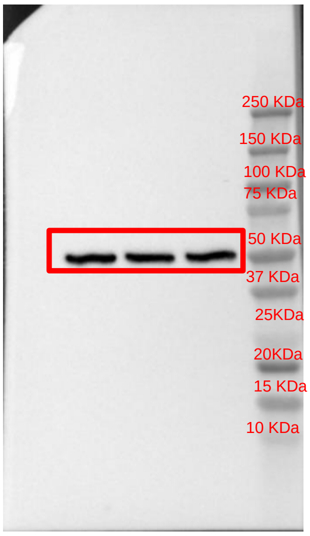

Lane 1 Mock
Lane 2 miR-195-5p mimic 30 nM
Lane 3 miR-195-5p mimic 50 nM
Lane 4 Standard Molecular Weight

Experiment 2 miR-195-5p mimic

KRT23

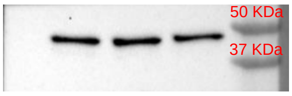

Lane 1 Mock
Lane 2 miR-195-5p mimic 30 nM
Lane 3 miR-195-5p mimic 50 nM
Lane 4 Standard Molecular Weight

ß-tubulin

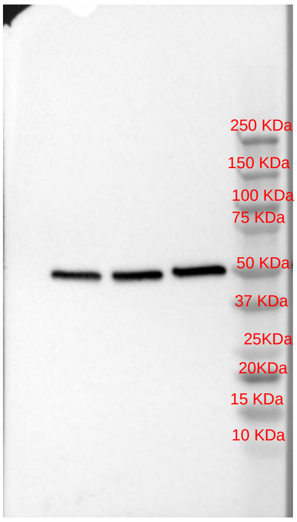

Lane 1 Mock
Lane 2 miR-195-5p mimic 30 nM
Lane 3 miR-195-5p mimic 50 nM
Lane 4 Standard Molecular Weight

Experiment 3+4 miR-195-5p mimic

KRT23

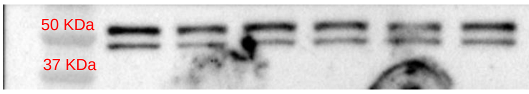

Lane 1 Standard Molecular Weight
Lane 2 Mock
Lane 3 miR-195-5p mimic 30 nM
Lane 4 miR-195-5p mimic 50 nM
Lane 5 Mock
Lane 6 miR-195-5p mimic 30 nM
Lane 7 miR-195-5p mimic 50 nM

ß-tubulin

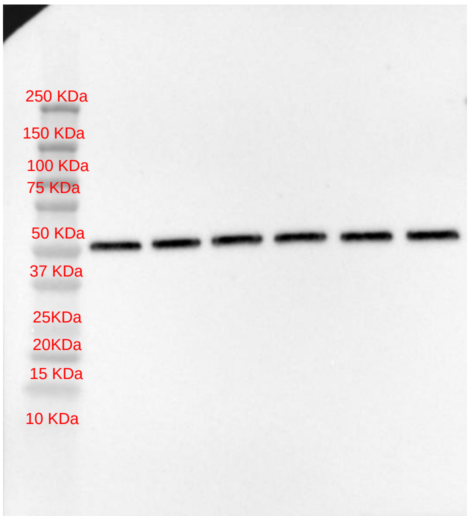

Lane 1 Standard Molecular Weight
Lane 2 Mock
Lane 3 miR-195-5p mimic 30 nM
Lane 4 miR-195-5p mimic 50 nM
Lane 5 Mock
Lane 6 miR-195-5p mimic 30 nM
Lane 7 miR-195-5p mimic 50 nM

Experiment 1 miR-195-5p inhibitor

KRT23

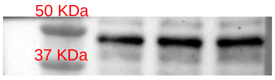

Lane 1 Standard Molecular Weight
Lane 2 Mock
Lane 3 miR-195-5p inhibitor 30 nM
Lane 4 miR-195-5p inhibitor 50 nM

ß-tubulin

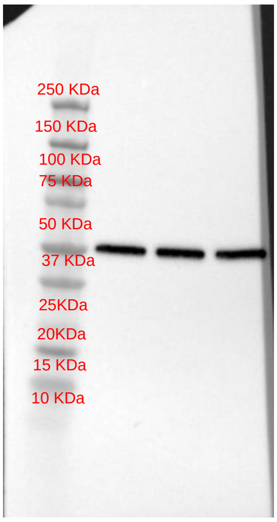

Lane 1 Standard Molecular Weight
Lane 2 Mock
Lane 3 miR-195-5p inhibitor 30 nM
Lane 4 miR-195-5p inhibitor 50 nM

Experiment 2 miR-195-5p inhibitor

KRT23

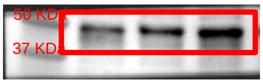

Lane 1 Standard Molecular Weight
Lane 2 Mock
Lane 3 miR-195-5p inhibitor 30 nM
Lane 4 miR-195-5p inhibitor 50 nM

ß-tubulin

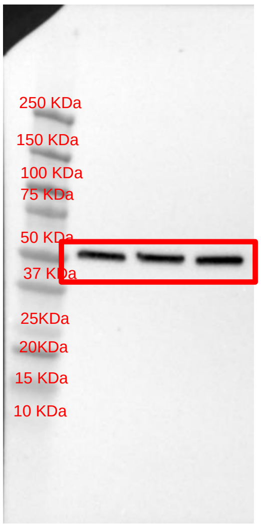

Lane 1 Standard Molecular Weight
Lane 2 Mock
Lane 3 miR-195-5p inhibitor 30 nM
Lane 4 miR-195-5p inhibitor 50 nM

Experiment 3 miR-195-5p inhibitor

KRT23

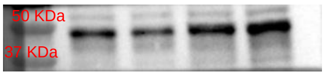

Lane 1 Standard Molecular Weight
Lane 2 Mock
Lane 3 Mock
Lane 3 miR-195-5p inhibitor 30 nM
Lane 4 miR-195-5p inhibitor 50 nM

ß-tubulin

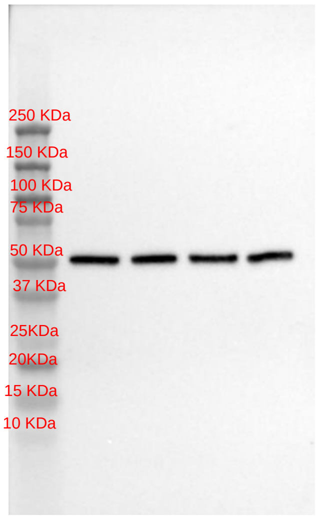

Lane 1 Standard Molecular Weight
Lane 2 Mock
Lane 3 Mock
Lane 3 miR-195-5p inhibitor 30 nM
Lane 4 miR-195-5p inhibitor 50 nM

Ht29 Western blot for KRT23

Experiment 1+2 miR-195-5p mimic

KRT23

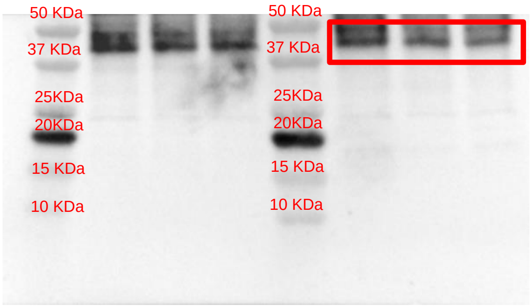

Lane 1 Standard Molecular Weight
Lane 2 Mock
Lane 3 miR-195-5p mimic 30 nM
Lane 4 miR-195-5p mimic 50 nM
Lane 5 Standard Molecular Weight
Lane 6 Mock
Lane 7 miR-195-5p mimic 30 nM
Lane 8 miR-195-5p mimic 50 nM

ß-tubulin

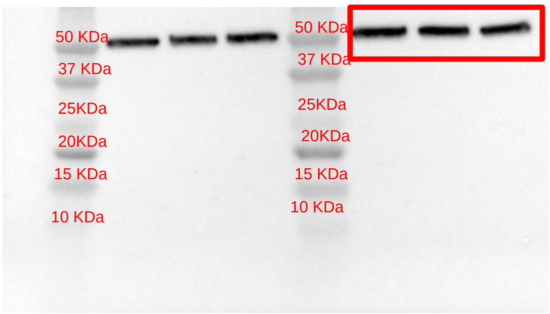

Lane 1 Standard Molecular Weight
Lane 2 Mock
Lane 3 miR-195-5p mimic 30 nM
Lane 4 miR-195-5p mimic 50 nM
Lane 5 Standard Molecular Weight
Lane 6 Mock
Lane 7 miR-195-5p mimic 30 nM
Lane 8 miR-195-5p mimic 50 nM

Experiment 3+4 miR-195-5p mimic

KRT23

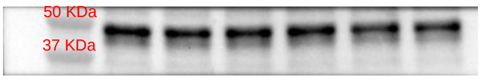

Lane 1 Standard Molecular Weight
Lane 2 Mock
Lane 3 miR-195-5p mimic 30 nM
Lane 4 miR-195-5p mimic 50 nM
Lane 5 Mock
Lane 6 miR-195-5p mimic 30 nM
Lane 7 miR-195-5p mimic 50 nM

ß-tubulin

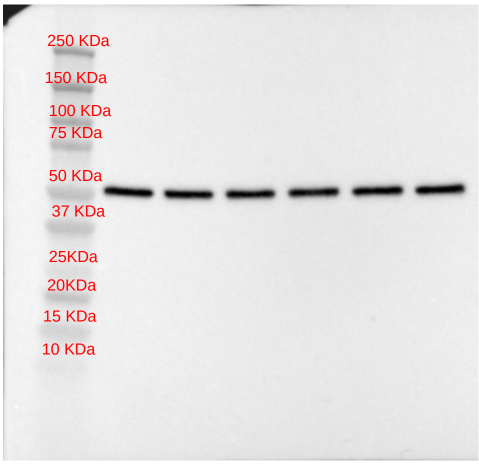

Lane 1 Standard Molecular Weight
Lane 2 Mock
Lane 3 miR-195-5p mimic 30 nM
Lane 4 miR-195-5p mimic 50 nM
Lane 5 Mock
Lane 6 miR-195-5p mimic 30 nM
Lane 7 miR-195-5p mimic 50 nM

Experiment 1 miR-195-5p inhibitor

KRT23

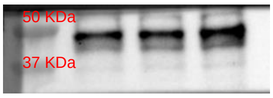

Lane 1 Standard Molecular Weight
Lane 2 Mock
Lane 3 miR-195-5p inhibitor 30 nM
Lane 4 miR-195-5p inhibitor 50 nM

ß-tubulin

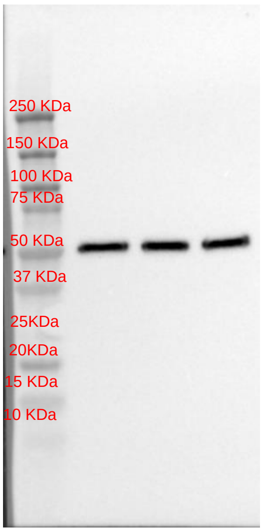

Lane 1 Standard Molecular Weight
Lane 2 Mock
Lane 3 miR-195-5p inhibitor 30 nM
Lane 4 miR-195-5p inhibitor 50 nM

Experiment 2 miR-195-5p inhibitor

KRT23

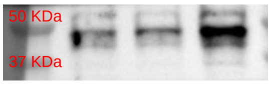

Lane 1 Standard Molecular Weight
Lane 2 Mock
Lane 3 miR-195-5p inhibitor 30 nM
Lane 4 miR-195-5p inhibitor 50 nM

ß-tubulin

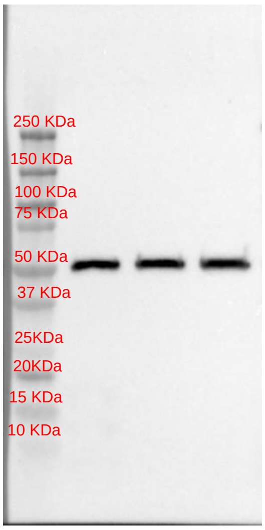

Lane 1 Standard Molecular Weight
Lane 2 Mock
Lane 3 miR-195-5p inhibitor 30 nM
Lane 4 miR-195-5p inhibitor 50 nM

Experiment 3 miR-195-5p inhibitor

KRT23

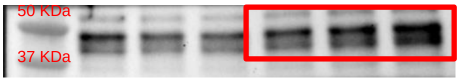

Lane 1 Standard Molecular Weight
Lane 2 Mock
Lane 3 miR-195-5p mimic 30 nM
Lane 4 miR-195-5p mimic 50 nM
Lane 5 Mock
Lane 6 miR-195-5p inhibitor 30 nM
Lane 7 miR-195-5p inhibitor 50 nM

ß-tubulin

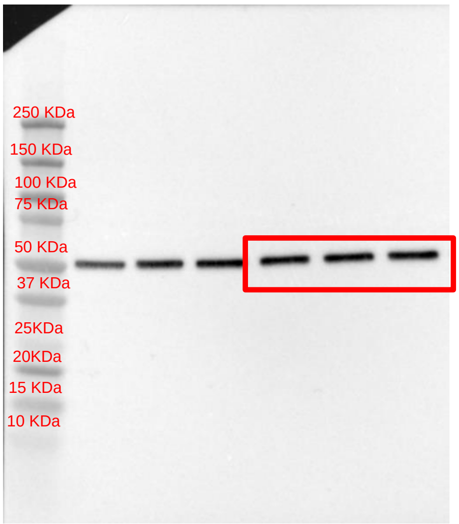

Lane 1 Standard Molecular Weight
Lane 2 Mock
Lane 3 miR-195-5p mimic 30 nM
Lane 4 miR-195-5p mimic 50 nM
Lane 5 Mock
Lane 6 miR-195-5p inhibitor 30 nM
Lane 7 miR-195-5p inhibitor 50 nM
