# Supplementary figures and images for: miR-195-5p Inhibits Colon Cancer Progression via KRT23 Regulation
Source: Pharmaceutics. 2024 Dec 4;16(12):1554. doi: 10.3390/pharmaceutics16121554 (PMC11680050; doi:10.3390/pharmaceutics16121554)

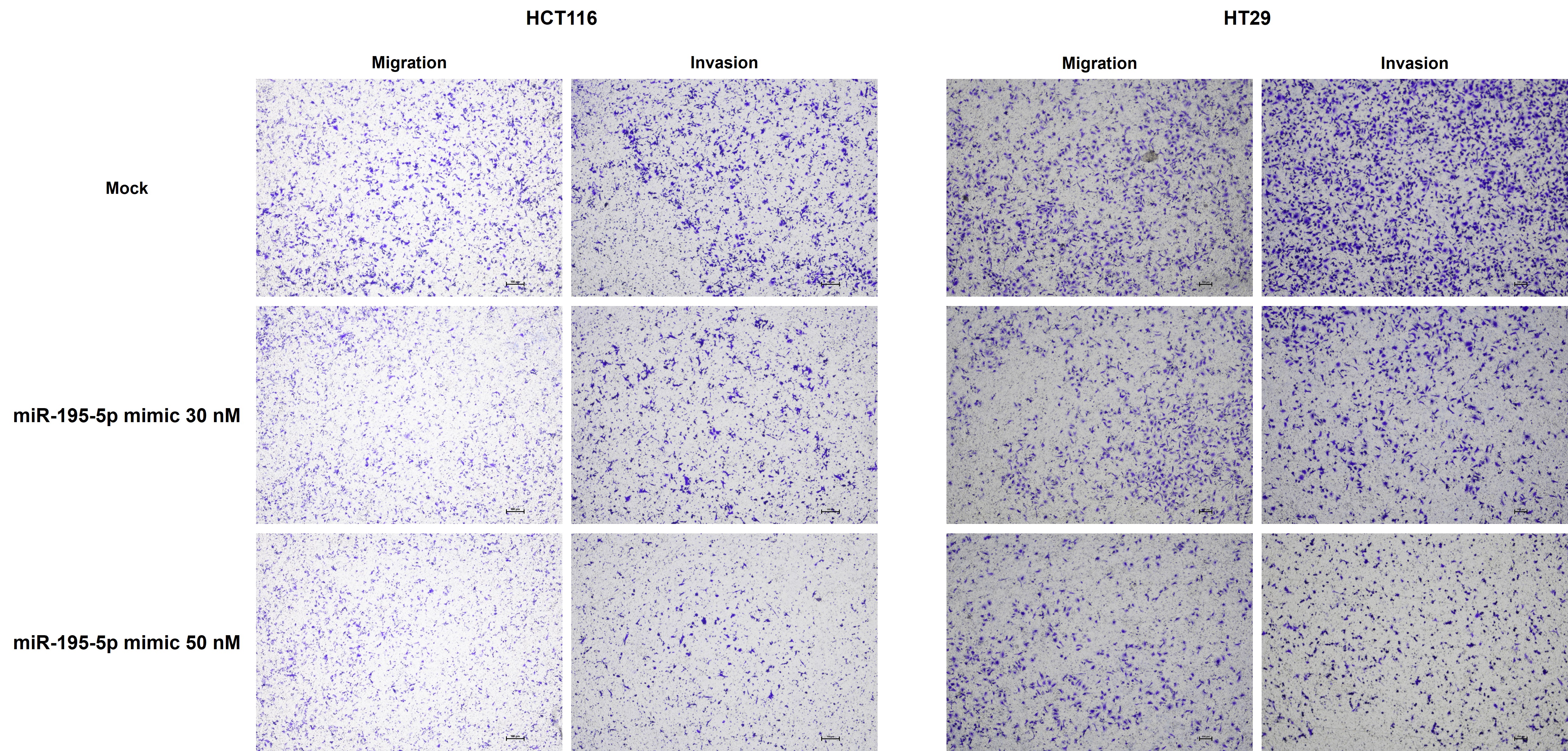

Supplement: Supplementary file 1 [file pharmaceutics-16-01554-s001.zip › Figure S1.jpg]

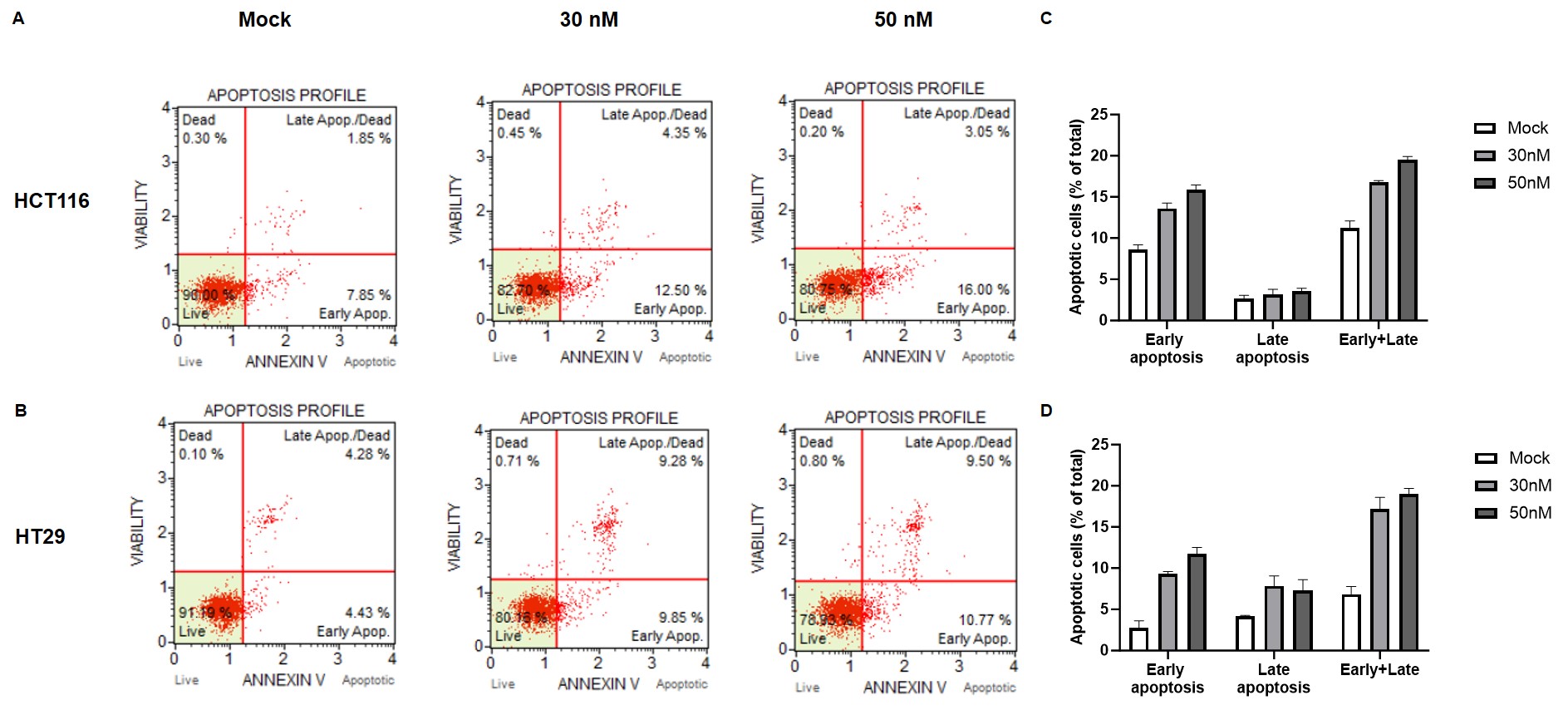

Supplement: Supplementary file 1 [file pharmaceutics-16-01554-s001.zip › Figure S2.jpg]
